# Tools to Measure Health Literacy among Adult Hispanic Populations with Type 2 Diabetes Mellitus: A Review of the Literature

**DOI:** 10.3390/ijerph191912551

**Published:** 2022-10-01

**Authors:** Carrie S. Standage-Beier, Shelby G. Ziller, Bahar Bakhshi, Oscar D. Parra, Lawrence J. Mandarino, Lindsay N. Kohler, Dawn K. Coletta

**Affiliations:** 1 School of Nutritional Sciences and Wellness, University of Arizona, Tucson, AZ 85721, USA; 2 Department of Epidemiology and Biostatistics, University of Arizona, Tucson, AZ 85721, USA; 3 UA Center for Disparities in Diabetes, Obesity and Metabolism, University of Arizona, Tucson, AZ 85724, USA; 4 Department of Medicine, Division of Endocrinology, University of Arizona, Tucson, AZ 85724, USA; 5 Department of Physiology, University of Arizona, Tucson, AZ 85724, USA

**Keywords:** health literacy, numeracy, Hispanic, type 2 diabetes, health promotion, health assessments

## Abstract

Health literacy (HL) is associated with short- and long-term health outcomes, and this is particularly relevant in Hispanics, who are disproportionally affected by lower HL. Hispanics have become the largest minority population in the United States. Also, Hispanics experience higher burdens of chronic diseases such as type 2 diabetes mellitus (T2DM) than non-Hispanic whites. Thus, effectively choosing culturally appropriate validated instruments that measure a marker found in health assessments should be a serious consideration. Using a systemized approach, we identified and reviewed 33 publications and found eight different HL and numeracy (separate or combined) instruments. We assessed the study designs and instrument structures to determine how HL was measured across these studies. We categorized the results into direct and indirect measurements of HL. The Test of Functional Health Literacy in Adults (TOFHLA) family of HL instruments was favored for direct measures of HL, while the Brief Health Literacy Screen (BHLS) instrument was favored for indirect measures. Despite identified trends in instruments used, more comprehensive measurement tools have been developed but not validated in Hispanic populations. In conclusion, further validation of more comprehensive HL instruments in adult Hispanic populations with T2DM could better assess HL levels and improve health promotion efforts.

## 1. Introduction

Lower levels of health literacy (HL) are associated with poorer health outcomes and the use of medical services [1,2]. HL, which has multiple conceptual frameworks, is one’s ability to obtain, comprehend, and interpret health and health-related concepts [3,4]. There are four widely referenced domains for HL; (1) functional HL, the ability to read and comprehend basic written text, write to complete forms, as well as locate and interpret health-related information; (2) interactive HL, to effectively speak and understand health-related information (communication); (3) critical HL, to navigate the health care system and make appropriate decisions about one’s health; and (4) numeracy HL, to interpret health-related numbers [3,5]. Being able to obtain, comprehend, and interpret health-related text could help influence an individual’s long-term health outcome. Measurement of HL, though, can be done on individual and community levels [6]. Assessment of HL at individual levels can occur in different settings, including public health and clinical environments [7]. Effectively measuring HL in different environments using culturally validated instruments is paramount.

Literature examining HL’s association with health outcomes in minority populations, such as Hispanic populations with T2DM, has grown. This research has become increasingly important as Hispanics have become the second largest United States minority population [8] and are disproportionally at-risk for several chronic conditions [9]. T2DM is one of these chronic conditions in which Hispanics have experienced a higher burden than non-Hispanic whites [10]. Chronic diseases reduce one’s quality of life, increase the risk for comorbid conditions and subsequently increase individual, local, and national healthcare financial responsibility [11]. Therefore, considerations should occur on improving the health assessments of populations disproportionally affected by chronic diseases, such as adult Hispanics with T2DM. 

Evidence suggests that HL associates with intermediate outcomes connected to personal health management, but HL is also an individual trait that is not exclusively shaped by either effective or ineffective health communications [12]. HL shows the interplay between the individual and their health care provider/system and community and the individual’s capacity to engage in skills found in the four HL domains [12]. As populations, including Hispanics but certainly not limited to, can experience both linguistic and cultural (cross-cultural) challenges when managing their health [13]. Neither the Spanish language nor cultural practices of Hispanic populations are monolithic; thus, providing tailored health assessments, education, and care to improve health outcomes is serious. In the Healthy People 2030 goals, HL has become a central focus to help reduce national health disparities and improve equity and health outcomes [14]. Thus, using appropriate HL measurement tools could provide more robust data that could help enhance preexisting and novel health promotion efforts. Therefore, critically assessing validated HL instruments utilized among Hispanic adults with T2DM could help improve future health assessment and promotion efforts.

The primary objective of this review was to critically assess HL instruments developed, validated, and used in studies with defined Hispanic populations through a systemized approach. Secondarily, we wanted to evaluate study designs and the use of individual or combined numeracy instruments used in conjunction with HL. We hypothesize that despite several decades of HL research, the development, validation, and use of culturally sensitive HL instruments in Hispanic populations with T2DM is limited.

## 2. Methods

We utilized a systemized approach to conduct this literature review [12]. Three databases, PubMed, CINAHL, and Embase, were included. The following search terms were applied to each database: type 2 diabetes mellitus, health literacy, numeracy, self-efficacy, self-management, Hispanic American, Mexican American, Latino, and blood glucose. Searches were not limited to any timeframe and were restricted to the English language and geographical location of the United States. All systematic reviews identified were searched for relevant publications missed in the literature search. Studies were selected if participants were (1) adults aged 18 years and over, (2) of a self-reported Mexican American, Hispanic, or Latino origin, and studies (3) with an emphasis on T2DM self-management and self-efficacy, (4) measuring HL via a validated instrument, and (5) with designs including, randomized and nonrandomized controlled trials, intervention trials, or observational studies (cross-sectional, cohort, case–control, or longitudinal). All literature selected were examined for validated HL and numeracy instrument structures and how the instruments were used.

## 3. Summary of Literature

We identified 33 publications that examined HL and/or numeracy with defined adult Hispanic populations with T2DM [15,16,17,18,19,20,21,22,23,24,25,26,27,28,29,30,31,32,33,34,35,36,37,38,39,40,41,42,43,44,45,46,47]. The systemized approach identified 14 articles for inclusion. Additionally, the systematic review by Al Sayah et al., which aimed to identify instruments used to measure HL and numeracy in individuals with T2DM, ref. [5] was found, and due to the content similarity, the references were searched. As a result, 19 papers were considered for inclusion from Al Sayah et al. [5].

These 33 unique publications used 8 different HL and numeracy (separate or combined) instruments. Instruments either directly or indirectly measured HL and/or numeracy. All identified instruments found were: Short Test of Functional Health Literacy in Adults (S-TOFHLA), Brief Health Literacy Screen (BHLS), Wide Range Achievement Test (WRAT-4), Diabetes Numeracy Test (DNT)-15 Latino, Diabetes Numeracy Test (DNT)-5, Subjective Numeracy Scale (SNS), Test of Functional Health Literacy in Adults (TOFHLA), and Health Literacy Assessment Using Talking Touchscreen Technology (Health LiTT). The DNT family of instruments is the only tool specifically utilized in populations with diabetes. The instruments in these studies were given to either monolingual (English or Spanish) or bilingual (English and Spanish) speaking participants.

### 3.1. Direct Measurements

HL can be assessed in two manners, the first of which directly measures HL and numeracy. Tests that directly assessed HL and/or numeracy asked participants questions about reading prose or arithmancy [5,48]. An example of a HL question layout would be having a participant read a passage and answer questions about the materials they had just read [49]. In comparison, numeracy-focused questions relate to understanding numeric information such as prescription bottles [49].

Direct measurement assessments are some of the oldest in use. The original WRAT assessment was published in 1946, with subsequent revisions coming in 1984 (WRAT-R), 1993 (WRAT 3), 2006 (WRAT 4), and 2017 (WRAT 5) [50,51]. The WRAT assesses an individual’s reading, sentence comprehension, spelling, and math computation [50,51]. This test uses age standardization methods to create scores. It can also standardize by percentiles and grade equivalencies, allowing for four forms of score distribution [5,50,51]. In literature examined, the WRAT-4 was used in two publications and specifically utilized for the math portion [44,45].

After the 1992 National Adult Literacy Survey findings were published, three primary HL tools were developed: REALM, TOFHLA, and S-TOFHLA [49,52,53,54]. The REALM instrument was created in 1993 and validated against the WRAT, Individual Achievement Test-Revised, and the Slosson Oral Reading Test-Revised [53]. This assessment was developed to determine reading and pronunciation skills in participants. Like the WRAT instruments, REALM presents grade equivalencies [5,53]. TOFHLA was created in 1995 with the intent of using actual hospital materials and creating a test for Spanish speakers [54]. Similar to other tools, the assessment was validated against WRAT-4 and REALM. TOFHLA’s evaluation determines participants reading comprehension and arithmancy skills [5,54]. S-TOFHLA was created in 1999 as a shorter version of the TOFHLA test [49], with a reduction from 17 numeracy items and 3 prose passages to 4 numeracy items and 2 prose passages [49]. Thus, the assessment is much quicker at 12 min rather than 22 min [5,49]. S-TOFHLA and TOFHLA present scores based on the inadequate, marginal, and adequate scales.

The TOFHLA family of instruments was preferred among the literature reviewed (Table 1). In the literature assessed, 20 of the 33 used an instrument in the TOFHLA family test, with 15 using the reading comprehension portion of the S-TOFHLA (Table 1). By focusing on assessing Hispanic participants, REALM and WRAT were likely not viable due to English language limitations.

The TOFHLA family of instruments is heavily relied upon, but due to the limited numeracy section, the DNT family of assessments was created [55]. This instrument directly assessed numeracy with diabetes-specific content [17,44,45,55]. The DNT family of instruments includes the DNT, DNT-15, DNT-15 Latino, and DNT-5 [45,55]. In addition, the DNT-15 Latino and the DNT-5 have explicitly been validated with Hispanic and Spanish-speaking participants [17,43,45]. These series of assessments allow for diabetes-specific numeracy assessments, which are critical for populations with T2DM.

As research and technology have progressed, newer HL tools have emerged. One such tool is the Health LiTT, initially created to be a multimedia test built with novel health information technology and psychometric principles [24,47]. This complex tool also utilized item response theory to create a scoring model. The assessment was validated in English and Spanish separately, with a Hispanic sample included in both validations [24,47]. Health LiTT qualifies as a TOFHLA family measure style but creates a modern delivery system [48]. The Health LiTT allows for a comprehensive assessment of both HL and numeracy and is a good representation of newer developments in instruments.

### 3.2. Indirect Measurements

In identified literature, 10 publications used the indirect instruments BHLS and SNS (Table 2). Indirect instruments have participants self-reflect on their HL and numeracy skills. An example from Nelson et al. [29] is: “How confident are you filling out medical forms by yourself?”. These assessments utilize Likert scales to quantify individuals’ self-assessments. Indirect instruments tend to be shorter and geared toward clinical settings [56,57]. For example, Chew et al. [56] developed the 3 question HL assessment (also referred to as BHLS) to allow physicians to quickly assess the patient’s HL. The BHLS was validated against S-TOFHLA and REALM in a Veteran Affairs (VA) outpatient population [56,57]. The BHLS was later validated for Spanish speakers and against the S-TOFHLA [35]. English and Spanish validations found the confidence question to be the best estimation of HL [35,57].

Indirect numeracy instruments were created with a similar intent; for example, in literature found, the indirect instruments were utilized in larger studies, conducted over the phone or through an interview, or had long surveys. The SNS is an 8 questions assessment of numeracy skills that utilizes Likert scales to quantify individuals’ self-assessments [58]. The English version of the SNS was validated against the Lipkus numeracy scale [58]. Spanish version of the SNS was validated against DNT-5 and SNS English version [17]. This instrument is easily conducted with participants in person or over the phone [17,58].

### 3.3. Studies Overview

Across all 33 publications, 22 were cross-sectional, 9 were intervention, and 2 were cohort studies. Within the cross-sectional studies, 10 used HL and numeracy as mediators or covariates [18,23,26,28,29,30,31,32,36], 7 used HL and numeracy as the primary exposure [15,24,27,32,37,44,46], and 5 were validation studies [17,35,38,45,47,52]. All 9 intervention studies assessed diabetes education or care programs and collected baseline HL and numeracy [16,21,22,39,40,41,42,43]. Both cohort studies examined HL and numeracy as the primary exposure [19,20].

All publications included Hispanic participants with T2DM. Five studies had 100% Hispanic participants [16,18,26,44,45], twenty had between 20% to 82%, and the remaining seven had 14% or less [19,20,25,27,28,29,32,52]. Of the articles with 100% Hispanic participants, three had mostly Mexican nationality [18,44,45], while the other two predominantly had Puerto Rican and Dominican nationality [16,26]. Of the remaining articles, 17 referred to participants as Hispanic [17,19,20,23,28,29,31,32,33,34,35,36,40,41,42,43,47], 8 referred to participants as Latino [15,21,22,25,30,37,38,39,46], and 2 used Hispanic/Latino [24,27].

## 4. Discussion

Through a systemized approach, this review identified and summarized 33 studies with a defined Hispanic population with T2DM. Also, it identified 8 validated HL and/or numeracy instrument structures. Our results showed that the TOFHLA family, notably the S-TOFHLA was the most used direct tool of HL instruments. S-TOFHLA was developed in 1999 by Baker et al. [49] and is a 36 self-reported question instrument (including 4 numeracy questions) that has been validated for assessing functional HL [5,49]. Previous literature assessing HL instruments with diabetes self-management outcomes in more ethnically diverse study samples was consistent with this finding [5,27,59]. In our identified studies, the percent of study populations being Hispanic varied from 4% to 100% of total samples. Fifteen studies used the S-TOFHLA, with publication dates ranging from 2002 to 2017.

S-TOFHLA is the short version of the full length TOFHLA HL instrument, which is 50 reading comprehension and 17 numeracy-related questions. TOFHLA and its Spanish version (TOFHLA-S) were developed/validated in 1995 by Parker et al. [54] in a California outpatient clinic sample of English (n = 200) and Spanish-speaking (n = 203) adults. This tool was shown to be reliable at measuring the ability of patients to read health-related information (i.e., functional HL) [54]. However, it is worth highlighting here that TOFHLA and S-TOFHLA do not assess interactive or critical HL, and the S-TOFHLA does not have enough comprehensive questions to ideally assess numeracy [5].

The two most used instruments in our reviewed studies were S-TOFHLA and BHLS. However, when considering those with chronic conditions such as T2DM, one’s level of numeracy should also be assessed to help measure their diabetes self-management abilities. Diabetes management includes self-monitoring blood glucose values, and long-term medications as they are needed to prevent severe/life-threatening health complications, which is worth noting [9,22]. Additionally, 6 studies used combined HL and numeracy tools, which were the TOFHLA [27,46], the S-TOFHLA [19,20], and Health LiTT [24,47]. Only one study combined indirect measures of HL and numeracy tools (BHLS and SNS) [17]. No identified articles had individual measurement tools for combined indirect HL and numeracy. At the same time, all three studies conducted by White et al. [43,44,45] combined HL via S-TOFHLA and either the WRAT-4, DNT-15 Latino, or DNT numeracy instruments. These general HL tools do not comprehensively assess numeracy concepts; thus, when trying to determine HL’s impact on diabetes self-management, a comprehensive assessment of numeracy should be considered.

Despite TOFHLA and S-TOFHLA’s development over 20 years ago and the BHLS almost 20 years ago, newer, more comprehensive HL measurement tools have been developed. One such tool is the Functional, Communicative, and Critical Health Literacy tool (FCCHL) which addresses all domains of HL but not numeracy. FCCHL was developed/validated in 2008 by Ishikawa et al. [60] in a sample of 138 outpatient Japanese adults with T2DM. This tool is a 14 self-reported item instrument that has since been validated in a 276 sample of midwestern United States adults with chronic comorbid conditions [61]. However, to our knowledge, the FCCHL has not been validated in Hispanic populations residing in the United States. Thus, further studies are needed to address this gap in the literature. Regarding the efficacy of using S-TOFHLA in minority populations, such as Hispanics, Chakkalakal et al. [17] previously tested the validity of various instruments, including S-TOFHLA instruments, in minority populations with T2DM [43]. In that study, Hispanics had significantly lower S-TOFHLA scores compared to non-Hispanic populations, suggesting a need for further evaluations of this instrument [43].

When examining instrument structures, scoring interpretations varied across identified studies. For example, the HL focused instrument S-TOFHLA had binary scores (incorrect, correct), and final score interpretations were inadequate, marginal, limited/low, and adequate. While the only other HL focused instrument was BHLS, the most common indirect measure of HL. Other associated studies interpreted scores as either percent of study population with low-moderate HL or mean scores. The combined HL/numeracy tool, TOFHLA, had the same scoring system/approach [27]. The only other identified combined HL/numeracy tools outside the TOFHLA family was the Health LiTT. WRAT-4 was the only instrument outside DNT-15 Latino and DNT (numeracy focused) instruments that scored using mean raw or mean standardized scores. The WRAT family of instruments, originally developed in 1946, have been revised for the fourth time, and both the REALM and TOFHLA family of instruments have been validated against various WRAT versions. Overall, WRAT instruments have been more commonly used in instrument validation than assessment.

Despite growing literature from smaller scale assessments of HL in Hispanic populations, limited work has been done nationally. The last national assessment of adult literacy in the United States which included Hispanics was the 2003 National Assessment of Adult Literacy (NAAL) survey. NAAL measured English literacy in non-Hispanic whites, blacks, Hispanics, and Asian/Pacific Islanders [62]. Not all Hispanics residing in the United States understand written or verbal English; therefore, the validity of the NAAL survey findings in Hispanics should be considered. Thus, critically examining literature published up to the time of our review was important for understanding current trends of validated instruments used to assess HL in Hispanic populations. It is worth noting that the Healthy People 2030 focuses on HL, which should hopefully spark more funding and research in this area [14].

Strengths of this study included having a systematic approach to examining validated HL instruments used in studies assessing HL in Hispanic adult populations with T2DM. In addition, multiple reviews of article screenings and data extractions were also performed. Notably, 19 of our included articles also appeared in Al Sayah et al. systematic review of HL and numeracy measures [5]. To be cohesive and provide updated literature specific to Hispanic populations, we used direct and indirect measures of HL/numeracy to communicate our results similarly to Al Sayah et al. [5]. From all identified studies, we could not determine HL’s impact on diabetes self-management due to the collinearity of multiple other determinants of health, such as acculturation and education [12]. In addition, determining culturally appropriate HL and/or numeracy tools was hindered due to the generalization of national origin. Only studies with 100% Hispanic populations were questioned and stratified by national origin. Those studies tended to be on the United States’ east coast. Additionally, studies predominately generalized Spanish as a monolithic language rather than accounting for different Spanish dialects. Overall, study findings strengthen the need for further validation of more comprehensive HL/numeracy tools in studies that account for acculturation. Nevertheless, our study’s findings will help guide future research performed in minority-focused biobanks, such as the El Banco por Salud, a predominately Hispanic population that is at-risk or has chronic diseases [9,63].

## 5. Conclusions

In summary, substantial work has been done developing and validating HL instruments over the past two decades. Further validation of more comprehensive HL instruments in adult Hispanic populations with T2DM could better assess HL levels and improve health promotion efforts and outcomes. This is an important area of further research that could ultimately help improve health outcomes of minority populations at-risk for chronic diseases, such as Hispanic adults.

## Figures and Tables

**Table 1:** Direct assessment health literacy and numeracy instruments found in publications having Hispanic populations with T2DM.

Measurements	Questions	Time (min)	Instrument Focus	Scoring
**Literacy Tools**				
Short Test of Functional Health Literacy in Adults (S-TOFHLA) [16,17,21,23,25,26,28,31,35,36,37,38,39,40,41,42]	36	7	Reading comprehension	Questions scored 0 or 1 based on correctness, partial correct answers are scored as incorrect. Scores presented as mean raw score (0–36) or score scale; 0–16: Inadequate, 17–22: Marginal, 0–22: Limited/Low, and 23–36: Adequate.
**Numeracy Tools**				
Wide Range Arithmetic Test (WRAT-4) [44,45]	55	20	Arithmetic computation	Questions scored 0 or 1 based on correctness then scores are converted into standard scores, percentiles, and grade equivalents based on provided materials. Scores presented as mean standardized score, percentiles, or math-grade equivalent; 4th grade or less, 5th–7th grade, and 8th–12th grade
Diabetes Numeracy Test (DNT)-15 Latino [44,45]	15	23	Diabetes-specific Arithmetic computation	Questions scored 0 or 1 based on correctness, partial correct answers are scored as incorrect. Scores presented as percent correct (0–100%).
Diabetes Numeracy Test (DNT)-5 [17,43]	5	11	Diabetes-specific Arithmetic computation	Questions scored 0 or 1 based on correctness, partial correct answers are scored as incorrect. Scores presented as percent correct (0–100%).
**Combined Literacy and Numeracy Tools**				
Test of Functional Health Literacy in Adults (TOFHLA) [27,46]	50 (HL *) 17 (N **)	22	Reading comprehension and numeracy test	Sum of the two sections added together. Numerical questions are weighted to equal 50 total points. Scores presented as mean raw score (0–100) or score scale; 0–59: Inadequate, 60–74: Marginal, and 75–100: Adequate.
Short Test of Functional Health Literacy in Adults (S-TOFHLA) [19,20]	36 (HL *) 4 (N **)	12	Reading comprehension and numeracy test	Questions scored 0 or 1 based on correctness, partial correct answers are scored as incorrect. Scores presented as mean raw score (0–100) or score scale; 0–53: Inadequate 54–66: Marginal, and 67–100: Adequate.
Health Literacy Assessment Using Talking Touchscreen Technology (Health LiTT) [24,47]	12 (HL *) 2 (N **)	18	Reading comprehension and numeracy test	Responses to multiple choice questions are either added together or IRT-based Bayesian expected a posteriori estimation response pattern was conducted. Scores presented as mean raw score or t score.

* HL = health literacy. ** N = numeracy.

**Table 2:** Indirect assessment of health literacy and numeracy instruments found in publications having Hispanic populations with T2DM.

Measurements	Questions	Timing	Instrument Focus	Scoring Calculation
**Literacy Tool**				
Brief Health Literacy Screen (BHLS) [15,17,22,29,30,31,32,33,34,35]	3	1.5 min	Self-reported HL skills	Responses to 5-point Likert scale questions added together. Scores presented as mean scores (3–15) or % of population with Low-moderate HL
**Numeracy Tool**				
Subjective Numeracy Scale (SNS) [17,22]	8	2 min	Self-reported numeracy skills	Responses to 6-point Likert scale questions added together. Scores presented as mean raw score (8–48) or mean response answer (1–6).

## Data Availability

No new data were created or analyzed in this study. Data sharing is not applicable to this article.
